# Peripheral CD39-expressing T regulatory cells are increased and associated with relapsing-remitting multiple sclerosis in relapsing patients

**DOI:** 10.1038/s41598-019-38897-w

**Published:** 2019-02-19

**Authors:** Nuria Álvarez-Sánchez, Ivan Cruz-Chamorro, María Díaz-Sánchez, Patricia Judith Lardone, Juan Miguel Guerrero, Antonio Carrillo-Vico

**Affiliations:** 1 Instituto de Biomedicina de Sevilla, IBiS (Universidad de Sevilla, HUVR, Junta de Andalucía, CSIC), Seville, Spain; 2 Departamento de Bioquímica Médica y Biología Molecular e Inmunología, Universidad de Sevilla, Seville, Spain; 3 Unidad de Gestión Clínica de Neurociencias, Servicio de Neurología del Hospital Universitario Virgen del Rocío, Seville, Spain; 4 Department of Clinical Biochemistry, Virgen del Rocío University Hospital, Seville, Spain

## Abstract

CD39, an ectonucleotidase that hydrolyses pro-inflammatory ATP, is a marker of highly active and suppressive T regulatory cells (Tregs). Although CD39 has a role in Treg suppression and might be important in the control of neuroinflammation in relapsing-remitting multiple sclerosis (RR-MS), to date, there are contradictory reports concerning the Tregs expression of CD39 in RR-MS patients. Thus, our objectives were to assess the activity and expression of CD39, especially in Tregs from peripheral blood mononuclear cells (PBMCs) of relapsing RR-MS patients compared with control subjects and to evaluate the association of CD39+ Tregs with disability and the odds of RR-MS. The activity and expression of CD39 and the CD39^+^ Treg frequency were measured in PBMCs from 55 relapsing RR-MS patients (19 untreated and 36 receiving immunomodulatory treatment) and 55 age- and sex-paired controls. Moreover, the association between CD39^+^ Tregs and RR-MS was assessed by multivariate logistic regression. CD39 activity and the frequency of CD39-expressing Tregs were elevated in relapsing RR-MS patients. Moreover, CD39^+^ Tregs were significantly correlated with the EDSS score and were independently associated with the odds of RR-MS. Our results highlight the relevance of CD39^+^ Treg subset in the clinical outcomes of RR-MS.

## Introduction

The pathogenesis of multiple sclerosis (MS), a chronic neuroinflammatory disease of the central nervous system (CNS), includes both inflammatory and neurodegenerative mechanisms which are triggered by the infiltration of myelin-specific CD4^+^ T helper (Th) cells. Th1 and Th17 subsets are considered of great importance in MS, because their signature cytokines are present in MS lesions^1,2^, and because activity and progression are associated with increased Th1 and Th17 responses in the cerebrospinal fluid of MS patients^3,4^.

The pathogenic Th1 and Th17 subsets can be controlled by T regulatory cells (Tregs)^5,6^, which are characterized by the expression of the nuclear transcription factor FoxP3, high levels of CD25 and low levels of CD127. Treg cells from MS patients are functionally impaired and show decreased suppressive and proliferative capacities^7,8^, which are partially recovered after immunomodulatory treatments^9,10^. In humans, a subset of highly suppressive Tregs expresses CD39 (ectonucleoside triphosphate diphosphohydrolase 1, E-NTPDase1). CD39 is a membrane protein that phosphohydrolyses ATP or ADP to yield AMP, which can be then hydrolysed to anti-inflammatory adenosine by CD73 (ecto-5′-nucleotidase, Ecto5′NTase)^11^. CD39-expressing Tregs are of special interest in MS research, as they are more stable^12^ and a have a higher proliferative, survival and suppressive capacities than do their CD39^−^ counterparts^12,13^. CD39^+^ Tregs can suppress both Th1 and Th17 responses in an adenosine-dependent manner, while CD39^−^ Treg cells only can suppress the Th1 response^14^. Human CD39^+^ Tregs have been described as regulatory effector/memory-like T cells^15^, and express higher levels of FoxP3, CD25, activation markers, co-inhibitory molecules and suppressive cytokines but lower levels of CD127 compared with CD39^−^ Tregs^12,14,16^.

There have been conflicting data regarding a possible alteration in CD39 expression by Treg cells in MS patients. In stable relapsing-remitting MS (RR-MS) patients, impairment of CD39 mRNA expression in peripheral blood mononuclear cells (PBMCs) has been shown^16,17^. But the frequency of CD39^+^ cells within the Treg population has been found to be reduced^15,16^. similar to^18^ or increased^19^ compared with that of healthy subjects. During MS exacerbations, the CD39 mRNA levels in PBMCs and CD39^+^ cell frequency within Tregs show either no differences^16,18^ or an increase^19^ in comparison with controls. Different immunomodulatory treatments such as interferon (IFN) β, fingolimod, alemtuzumab and corticoids have been reported to increase the expression and levels of CD39, the frequency of CD39^+^ Treg cells, and the ATP/ADP hydrolysis capacity of those cells^15,17,19–22^. Moreover, CD39^+^ Tregs isolated from RR-MS patients have been shown to have impaired suppressive activity over the Th17 response^14^.

Thus, the objective of our work was to analyse the expression of CD39 in PBMCs from relapsing RR-MS patients and age- and sex-paired healthy subjects, with a special focus on the expression of CD39 on Treg cells.

## Results

### RR-MS patients show an elevated CD39 ecto-ATPase activity

**Figure 1:**
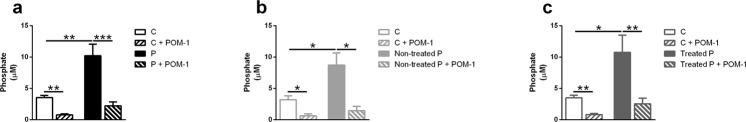
Ecto-ATPase activity is increased in PBMCs from relapsing-remitting multiple sclerosis patients regarding controls. Levels of inorganic phosphate released by the hydrolysis of ATP by ecto-ATPases present in PBMCs from all controls (C) and patients (P) (n = 17) (**a**) patients who were not receiving immunomodulatory drugs and their age- and sex-paired controls (n = 6) (**b**) and patients treated with immunomodulatory therapies and their age- and sex-paired controls (n = 11) (**c**). PBMCs from all subjects were also incubated with the CD39 inhibitor POM-1 to confirm that the ecto-ATPase activity assessed was due to CD39. Data represent the means ± SEM. *p ≤ 0.05; **p ≤ 0.01; ***p ≤ 0.001.

PBMCs from patients and controls were incubated with ATP and the ecto-ATPase activity was assessed by measuring the amount of inorganic phosphate released in culture supernatants. Independently of immunomodulatory drug treatment status, PBMCs from MS patients showed a higher ecto-ATPase activity than did the controls (Fig. 1a–c). The ecto-ATPase activity measured was significantly inhibited by the CD39 inhibitor POM-1 in both patients and controls (Fig. 1a–c).

### Expression of CD39 is increased in Tregs from RR-MS patients

**Figure 2:**
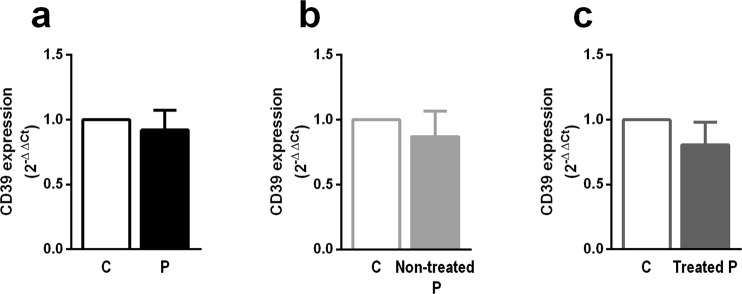
CD39 mRNA levels in PBMCs are not changed in relapsing-remitting MS patients (P) compared with controls (C). CD39 mRNA levels in PBMCs from all patients and controls (n = 28) (**a**) patients who were not receiving immunomodulatory treatments and their age- and sex-paired controls (n = 11) (**b**) and patients who were receiving immunomodulatory treatments and their age- and sex-paired controls (n = 17) (**c**). Data represent the means ± SEM. *p ≤ 0.05.

**Figure 3:**
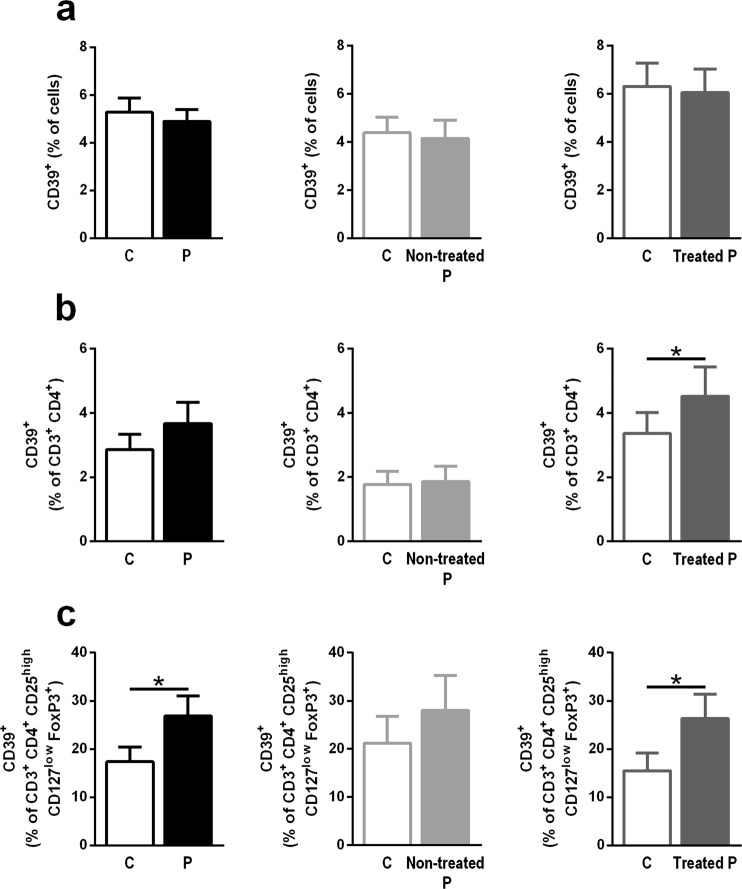
Increased frequency of peripheral CD39-expressing Tregs in relapsing-remitting MS patients (P) compared with controls (C). (**a**) Frequency of CD39^+^ total cells in all patients and controls (n = 46) (left; black); untreated patients and their controls (n = 15) (middle; light grey); and treated patients and their controls (n = 31) (right; dark grey). (**b**) Frequency of CD39-expressing CD3^+^ CD4^+^ T cells in all patients and controls (n = 46) (left; black); non-treated patients and their controls (n = 15) (middle; light grey); and treated patients and their controls (n = 31) (right; dark grey). (**c**) Frequency of CD39^+^ CD3^+^ CD4^+^ CD25^high^ CD127^low^ FoxP3^+^ Treg cells in all patients and controls (n = 46) (left; black); untreated patients and their paired controls (n = 15) (middle; light grey); and treated patients and their controls (n = 31) (right; dark grey). Data represent the means ± SEM. *p ≤ 0.05.

CD39 mRNA expression was not found to be altered in PBMCs from either RR-MS patients who were treated with immunomodulatory drugs or those who were not treated, compared with controls (Fig. 2a–c). The frequency of CD39^+^ total cells was also not altered (Fig. 3a). However, a trend towards an increased frequency of CD3^+^ CD4^+^ T cells expressing CD39 was observed on MS patients (Fig. 3b left; p = 0.069); when MS patients were grouped according to their treatment status, only treated MS patients showed a significantly higher frequency of CD39-expressing CD3^+^ CD4^+^ T cells than did the age- and sex-paired controls (Fig. 3b middle and right).

The assessment of CD39^+^ cells presence specifically in the Tregs population showed an increased frequency of CD39^+^ cells within the CD3^+^ CD4^+^ CD25^high^ CD127^low^ FoxP3^+^ subset from RR-MS patients compared with controls; this increase was only statistically significant in the patients who underwent immunomodulatory treatment (Fig. 3c).

### The frequency of CD39^+^ Treg cells correlates with RR-MS disability and is associated with MS

The correlations between the frequency of CD39^+^ cells in the CD3^+^ CD4^+^ CD25^high^ CD127^low^ FoxP3^+^ Treg subset and the EDSS score, pro- and anti-inflammatory responses and ecto-ATPase activity in controls and patients are shown in Table 1, while Table 2 shows correlation between the MFI of CD39 in the CD39-expressing Tregs and pro-inflammatory responses. In healthy subjects, the frequency of CD39^+^ Tregs showed a positive correlation with both the interleukin (IL) 10/IFNγ ratio and the ecto-ATPase activity, and a negative correlation with the frequency of Th1 cells. However, in MS patients all of these correlations were non-significant, and the frequency of CD39-expressing Treg cells was significantly correlated with the EDSS score, the MFI of IFNγ in Th1 cells and the levels of IL-17A measured in PBMC culture supernatants. With regard to the surface expression of CD39 in the CD3^+^ CD4^+^ CD25^high^ CD127^low^ FoxP3^+^ CD39^+^ subset, it significantly correlated with IFNγ and IL-17A levels, and with the MFI of TNF in CD4^+^ TNF^+^ cells in both patients and controls, and also with the MFI of IFNγ in CD4^+^ IFNγ^+^ cells and the frequency of CD4^+^ IL-17A^+^ cells only in MS patients (Table 2).

**Table 1:** Non-parametric correlations among the frequency of CD39^+^ cells in the CD3^+^ CD4^+^ CD25^high^ CD127^low^ FoxP3^+^ population and EDSS score (only in patients), Th1 and Th17 effector, and regulatory responses, and CD39 ecto-ATPase activity.

		EDSS score	Frequency of IFNγ^+^ CD4^+^ cells	IFNγ MFI in IFNγ^+^ CD4^+^ cells	IL-17A levels (pg/ml)	IL-10/IFNγ ratio	Ecto-ATPase activity
Controls	r	N/A	−0.36*	0.19	0.22	0.34*	0.77*
Patients	r	0.63**	−0.02	0.42**	0.36*	0.05	−0.14

Spearman’s correlation coefficient (r) between frequency of CD39^+^ cells in the CD3^+^ CD4^+^ CD25^high^ CD127^low^ FoxP3^+^ population and the variables stated. N/A: not available.

*p ≤ 0.05; **p ≤ 0.01.

**Table 2:** Non-parametric correlations among the MFI of CD39 in cells in the CD3^+^ CD4^+^ CD25^high^ CD127^low^ FoxP3^+^ CD39^+^ population and Th1 and Th17 effector responses.

		IFNγ levels (pg/ml)	IFNγ MFI in IFNγ^+^ CD4^+^ cells	TNF MFI in TNF^+^ CD4^+^ cells	IL-17A levels (pg/ml)	Frequency of IL-17^+^ CD4^+^ cells
Controls	r	0.35*	0.25	0.37*	0.47**	0.00
Patients	r	0.31*	0.40**	0.45**	0.49***	0.34*

Spearman’s correlation coefficient (r) between frequency MFI of CD39 in cells in the CD3^+^ CD4^+^ CD25^high^ CD127^low^ FoxP3^+^ CD39^+^ population and the variables stated.

*p ≤ 0.05; ** p≤ 0.01; ***p ≤ 0.001.

A multivariate logistic regression analysis was performed to evaluate the association between the frequency of CD3^+^ CD4^+^ CD25^high^ CD127^low^ FoxP3^+^ Treg cells expressing CD39 and RR-MS prevalence, adjusted for age and sex. The frequency of CD39^+^ Tregs was significantly associated with increased odds of RR-MS; the odds ratio (OR) for RR-MS per one-unit increase in the frequency of CD39-expressing Tregs was 1.03 (95% CI, 1.01–1.06; p = 0.005).

## Discussion

Our study shows that, during a relapse, CD39 ecto-ATPase activity is increased in RR-MS patients, independently of immunomodulatory treatment status, and the CD39 levels are increased specifically in circulating CD4^+^ CD25^high^ CD127^low^ FoxP3^+^ Treg cells from RR-MS patients compared with sex- and age-paired heathy subjects, especially in treated RR-MS patients. Moreover, we show, for the first time, that the frequency of CD39-expressing Treg cells correlates with disability in RR-MS patients and is significantly associated with the odds of RR-MS.

Ecto-ATPase activity was significantly elevated in RR-MS patients compared with healthy subjects, independently of immunomodulatory treatment status. To our knowledge, the only study to compare the ecto-ATPase activity of PBMCs from RR-MS patients with controls was performed on a mixed population of stable RR-MS patients who were untreated or treated with IFNβ. This study found that ecto-ATPase activity was increased in RR-MS patients^22^. A longitudinal study on a cohort of relapsing RR-MS patients, both treated and untreated, found that CD39 activity was increased after four days of corticoid treatment^19^; however, comparison was between before and after corticoid treatment, but not with healthy subjects. Thus, ours is the first work to report increased CD39 ecto-ATPase activity in PBMCs from relapsing RR-MS patients, and this increase occurs regardless of whether the patients are receiving immunomodulatory treatment.

We found that CD39 expression was specifically increased in Treg cells from relapsing RR-MS patients, and that this increase of CD39 levels was not found in other cell subsets as in all PBMCs or in all CD4^+^ T lymphocytes. Previous data regarding this issue in RR-MS patients showed contradictory results. Prior studies have reported either a general increase in CD39 expression (at the mRNA levels^19,20^ and an increased frequency of CD39^+^ cells in total PBMCs^22^) and lower^16^ or similar^16^ levels of CD39 expression to those of healthy controls. Similarly, the frequency of CD39^+^ cells among the Treg subset has been shown to be higher than^19,20^, similar to^15,16,18^ or lower^14–16^ than what is found in controls. These discrepancies could be attributed to differences in the phase and severity of the disease. Thus, while all our patients were experiencing a relapse, decreases in CD39 mRNA expression and CD39^+^ Tregs were reported in stable RR-MS patients^15,16^, and some studies reporting increases in general CD39 levels did not state whether the patients were in relapse or in remission^20,22^. Since it has been shown that CD39 levels can change between relapsing and stable phases of RR-MS^16^, these factors should be considered when comparing studies. Only three studies have been performed in relapsing RR-MS patients, and these studies showed that CD39 expression in PBMCs from RR-MS patients was similar to^16^ or higher^19^ than that in healthy subjects, and that the frequency of CD39-expressing Treg cells was increased^19^ or did not change^16,18^ in RR-MS patients compared with controls. These discrepancies might also have been due to the differing approaches used to identify Treg subsets. As recommended^23^, we have used the most stringent Treg definition (CD3^+^ CD4^+^ CD25^high^ CD127^low^ FoxP3^+^), in contrast to previous studies assessing this topic in relapsing RR-MS patients. These previous studies defined Treg cell subsets using either CD4^+^ CD25^high^ ^16^, CD4^+^ CD25^high^ FoxP3^+^ ^19^ or CD4^+^ CD25^high^ CD127^low^ ^18^ classifications; therefore, these studies could have included non-Treg cells (e.g., recently activated T effector cells) in their definition of the Treg subset.

Here, we show that the increased frequency of CD39-expressing Treg cells was only statistically significant in treated patients. On the one hand, this observation might account for some of the differences mentioned above, because the previous works included either only untreated patients^15,16^ or a mixed cohort of patients who were treated or untreated^14,18,19,22^, but failed to group the patients according to their treatment status. On the other hand, our results agree with several works showing that various immunomodulatory treatments increase CD39 expression in peripheral Treg cells from RR-MS patients, including corticoids^19^, fingolimod^17,20^, alemtuzumab^21^ and IFNβ^15^. In addition, it has been proposed that adenosine generation might contribute to the beneficial effects of IFNβ in MS^24^.

Even when immunomodulatory treatments increased CD39 expression in T lymphocytes and in Treg cells from RR-MS patients, CD39 activity was increased in PBMCs from both treated and untreated patients with respect to healthy subjects. This might be due to the effects of T cell receptor (TCR) ligation on CD39 activity, since it only becomes catalytically active in Tregs after TCR activation^15^, which would be expected in circulating Treg cells from RR-MS patients suffering an exacerbation, but not in healthy subjects.

While we found that the frequency of CD39-expressing Treg cells in control subjects correlated with a decreased Th1 response and an anti-inflammatory/pro-inflammatory ratio skewed towards a more protective cytokine milleu; these correlations were not found in patients, which could have been due to the partial functional impairment in CD39^+^ Tregs described previously, since MS CD39-expressing Tregs are able to suppress effector cell proliferation^16^ and some T effector responses, but not others^14^. Interestingly, the surface expression of CD39 in CD39^+^ Tregs showed a positive correlation with pro-inflammatory markers both in patients and controls. Although a possible pro-inflammatory effect of CD39 cannot be dismissed from our analysis, these results are probably due to pro-inflammatory molecules increasing CD39 expression; accordingly, pro-inflammatory signals, as TLR2 ligation^25^ and IL-6^26^, as well as TCR ligation^27^ have been shown to induce CD39 expression in T cells. Importantly, the frequency of CD39-expressing Treg cells was positively correlated with the disability of patients and was independently associated with the odds of suffering RR-MS. This effect might be an attempt to control the neuroinflammation in the long term and, perhaps, to compensate for the functional deficits reported in RR-MS Treg cells^7,8^.

CD39 is an important molecule in the Treg subset. This marker identifies Treg cells that are highly active^12,16^ and suppressive^12,13^, produce increased levels of the anti-inflammatory cytokine IL-10^12^ and show an increased migratory capacity towards the CNS^25,28^. Moreover, CD39 activity is very important for Treg function. Not only does the adenosine generated together by CD39 and CD73 participate in controlling Th1 and Th17 responses^14,29^, but it also attracts dendritic cells to Tregs, thus hampering the activation of T effector cells^30^. Additionally, Tregs from CD39^−/−^ mice cannot suppress the proliferation of T effector cells^11^. Furthermore, extracellular ATP, which is abundantly released in inflamed sites^31^, is particularly toxic to Treg cells^32^ and destabilizes their FoxP3 expression while promoting their transconversion into pathogenic Th17^33^.

The increase in CD39 expression in Tregs and in CD39 activity in PMBCs during a RR-MS exacerbation showed here might be an attempt to counterbalance the partial dysfunction of Treg cells in MS patients^7,8^, by mobilizing activated CD39^+^ Tregs from the lymphoid organs, showing an increased CD39 expression and activity due to the pro-inflammatory status found during a relapse in a MS patient. This mobilization would cause a subsequent elevation of both CD39 frequency and activity in the blood while on their way to the CNS. Those cells would then infiltrate the inflamed CNS where the increased CD39 activity might, on one hand, reduces the pro-inflammatory effects of extracellular ATP, while, on the other, it might help protect Treg cells from ATP-driven death and stabilize their phenotype and functionality in the inflamed CNS, allowing them to better control the neuroinflammatory process during the relapse.

In conclusion, we here report that relapsing RR-MS patients show an increase in both the CD39 ecto-ATPase activity in their PBMCs and the frequency of peripheral CD39^+^ Treg cells with respect to healthy controls. We also show, for the first time, that CD39-expressing Treg cells are associated with the disease, thus highlighting the importance of CD39 Tregs in RR-MS.

## Methods

### Patients

Fifty five RR-MS patients suffering an acute exacerbation were recruited from the Department of Neurology of the Virgen del Rocio University Hospital between 2011 and 2014 (Table 3). As shown in Table 3, 36 of the 55 patients were receiving immunomodulatory treatment when they suffered the relapse; treated patients were receiving mostly IFNβ (55.6%), but also glatiramer acetate (22,2%), natalizumab (11,1%) or fingolimod (11,1%) treatment. The study followed the Helsinki Declaration for medical research involving human subjects, it was approved by the Virgen del Rocío University Hospital ethical review board (reference number 14/2010) and a written informed consent was obtained from all study participants. Peripheral blood samples were taken from patients before receiving corticoid treatment, and from sex- and age-paired healthy donors on Vacutainer CPT tubes (BD). PBMCs were isolated by centrifugation and cultured at a density of 1 × 10^6^ cells/ml with RPMI-1640 supplemented with 10% foetal bovine serum, 2 mM L-glutamine and 50 U/ml of penicillin/streptomycin (all from BioWest). To measure cytokine production, cells were incubated for 48 hours with 8 µg/ml phytohemagglutinin (PHA; Sigma-Aldrich).

**Table 3:** Characteristics of relapsing-remitting multiple sclerosis (RR-MS) patients and healthy controls included in this study.

	Controls	RR-MS patients		
		All	Non-treated	Treated
Number of participants, n	55	55	19 (34.5%)	36 (65.5%)
Gender, women/men, n [%]	41/14 [74.5/25.5]	41/14 [74.5/25.5]	16/3 [84.2–15.8]	25/11 [69.4–30.6]
Age at study, years (mean [95% CI])	36.4 [34.1–38.6]	36.1 [33.8–38.4]	35.8 [31.4–40.2]	36.3 [33.4–39.1]
Disease progression, years (mean [95% CI])		6.5 [4.9–8.1]	6.1 [3.4–8.9]	6.7 [4.7–8.7]
EDSS (mean [95% CI])		3.7 [3.2–4.3]	3.5 [2.3–4.7]	3.8 [3.1–4.5]
Frequency of IFNγ^+^ CD4^+^ cells (mean [95% CI])	1.9 [1.3–2.4]	1.9 [1.2–2.5]	2.0 [0.6–3.3]	1.8 [1.1–2.6]
IFNγ MFI in CD4^+^ IFNγ^+^ (mean [95% CI])	3253.6 [2403.5–4103.7]	2672.7* [1892.9–3452.5]	3259.9 [1934.4–4585.4]	2261.7 [1253.9–3269.4]
TNF MFI in CD4^+^ TNF^+^ (mean [95% CI])	3221.0 [2482.0–3960.0]	3598.0* [2569.6–4626.3]	4441.2 [1999.7–6882.6]	3049.9 [2232.0–3867.9]
Frequency of IL-17^+^ CD4^+^ cells (mean [95% CI])	0.4 [0.2–0.5]	0.3 [0.2–0.4]	0.4 [0.2–0.6]	0.3 [0.1–0.4]
IL-10 (pg/ml) (mean [95% CI])	666.8 [467.3–866.3]	441.2* [287.1–595.3]	408.6 [136.5–680.7]	458.5 [260.9–656.1]
IFNγ (pg/ml) (mean [95% CI])	2750.1 [1963.3–3636.9]	1999.6 [1173.7–2825.6]	2038 [406.7–3669.6]	1979.2 [984.9–2973.5]
IL-17A (pg/ml) (mean [95% CI])	279.0 [150.2–407.9]	458.0 [166.4–749.7]	469.4 [233.0–705.7]	450.3 [0–934.0]
IL-10/IFNγ ratio (mean [95% CI])	0.8 [0.4–1.3]	1.6 [0.4–3.2]	0.8 [0–1.6]	2.1 [0–4.5]

All values are expressed as a mean and 95% confidence interval (95% CI) or as percentage. The characteristics of the subjects were compared between controls and patients using the Wilcoxon signed-rank test (for continuous variables) or the Pearson’s χ^2^ test (for categorical variables). *p < 0.05 vs. controls.

### RNA extraction, retro-transcription and real-time quantitative PCR

RNA was extracted from *ex vivo* PBMCs using TriPure Isolation Reagent according to the manufacturer’s instructions, and retro-transcribed to cDNA with Transcriptor First Strand cDNA Synthesis Kit. Real-time qPCR was performed on a LightCycler 480 using LightCycler 480 SYBR Green I Master (all from Roche Diagnostic). The primer sequences are detailed in Supplemental Table 1. The relative expression levels were calculated using the 2^−ΔΔCt^ method, and normalized to the expression of β-actin. The stability of the expression of β-actin across different samples and groups was tested by comparing the C_t_ values in the control group with those in the patients (all patients group), the non-treated patients and the treated patients. No significant differences were found in the expression of β-actin across the different groups.

### Flow cytometry

Non-stimulated PBMCs were stained for CD3, CD4, CD25, CD127, and CD39, were then fixed and permeabilised using the FoxP3 Staining Buffer Set (eBioscience), followed by the intracellular staining of FoxP3 (see Supplemental Table 2 for flow cytometry antibody characteristics).

The intracellular production of pro-inflammatory cytokines by PBMCs was measured in PHA-stimulated PBMCs incubated with brefeldin A (eBioscience) for the last 5 hours of culture. The cells were stained for extracellular CD4, permeabilized with BD Cytofix/Cytoperm kit (BD Biosciences), and stained for the intracellular IFNγ, TNF and IL-17 (see Supplemental Table 2 for flow cytometry antibodies characteristics). FACS analysis was carried out using a LSRFortessa (BD Biosciences) and data were analyzed using FlowJo software (Treestar).

### Cytokine quantification

Representative pro- and anti-inflammatory cytokines were measured in supernatants from 48-hour-, PHA-stimulated PBMC cultures using a fluorescent bead assay (13plex FlowCytomix kit, eBioscience) following the manufacturer’s instructions. Median fluorescence intensity (MFI) data were analysed using the FlowCytomix Pro 2.4 software (eBioscience).

### Ecto-nucleotidase activity assay

Non-stimulated PBMCs were washed three times with phosphate-free buffer (0.5 mM CaCl_2_, 120 mM NaCl, 5 mM KCl, 50 mM Tris-HCl, pH 8), resuspended in phosphate-free buffer in the presence or absence of 250 µM ATP (Sigma-Aldrich) and then incubated at 37 °C for 30 minutes. The ATP hydrolysis was stopped by placing cells on ice for 10 minutes. Then, 80 µl of the supernatants were used to measure inorganic phosphate using the Malachite Green Phosphate Assay Kit (BioChain), according to the manufacturer’s instructions. To exclude the possibility that non-enzymatic degradation of ATP and the direct release of phosphate by PBMCs contributed to the phosphate levels measured, controls using ATP incubated without cells and cells incubated without ATP were included, respectively. To determine whether the inorganic phosphate was selectively produced by CD39 activity, the NTPDase inhibitor POM-1 (10 µM; Tocris) was used.

### Statistical analysis

All results are expressed as the mean ± SEM. Data were analysed with the SPSS v24.0 software (IBM), using the Wilcoxon signed-rank test for comparing controls and patients, since none of the variables were normally distributed; Spearman’s rho correlation coefficient test was performed to analyse correlations between the frequency of CD39^+^ cells in the Treg population and disease characteristics and immune responses; and multivariate logistic regression to assess the association between this subset and prevalent MS. The analyses were performed by comparing all of the patients with their controls and by grouping them into non-treated (n = 19) and treated (n = 36) groups with their respective controls. Values of p ≤ 0.05 were considered to be statistically significant.

## Supplementary information

Supplemental Table 1 and Table 2

## Data Availability

The datasets used and/or analyzed during the current study are available from the corresponding author on reasonable request.
