# Supplementary material for: Peripheral CD39-expressing T regulatory cells are increased and associated with relapsing-remitting multiple sclerosis in relapsing patients
Source: Sci Rep. 2019 Feb 19;9:2302. doi: 10.1038/s41598-019-38897-w (PMC6381140; doi:10.1038/s41598-019-38897-w)
Supplement: Supplementary file 1 — Supplemental Table 1 and Table 2 [file 41598_2019_38897_MOESM1_ESM.docx]

**Peripheral CD39-expressing T regulatory cells are increased and associated with relapsing-remitting multiple sclerosis in relapsing patients**

Nuria Álvarez-Sánchez^a^, Ivan Cruz-Chamorro^a,b^, María Díaz-Sánchez^c^, Patricia Judith Lardone^a,b^, Juan Miguel Guerrero^a,b,d^, Antonio Carrillo-Vico^a,b,*^

^a^ Instituto de Biomedicina de Sevilla, IBiS (Universidad de Sevilla, HUVR, Junta de Andalucía, CSIC), Seville, Spain.

^b^ Departamento de Bioquímica Médica y Biología Molecular e Inmunología, Universidad de Sevilla, Spain.

^c^ Unidad de Gestión Clínica de Neurociencias, Servicio de Neurología del Hospital Universitario Virgen del Rocío, Seville, Spain.

^d^ Department of Clinical Biochemistry, Virgen del Rocío University Hospital, Seville, Spain.

Address correspondence and reprint requests to: Antonio Carrillo-Vico, Instituto de Biomedicina de Sevilla, IBIS (Universidad de Sevilla, HUVR, Junta de Andalucía, CSIC). Avda. Manuel Siurot s/n, 41013 Seville, Spain; Tel.: +34955923106; Fax: +34954907048; E-mail: [vico@us.es](mailto:vico@us.es)

**Supplemental Table 1.** Sequences of the primers and real-time qPCR conditions used in this study.

| **Gene** |  | **Sequence** | **ng of cDNA/well** | **Annealing temperature** |
| --- | --- | --- | --- | --- |
| *CD39* | Forward | 5’-TGTGCTGGATGCGGGTTC-3’ | 80 | 56°C |
|  | Reverse | 5’-TGCCGTGGCTCCCAGGTAAAC-3’ |  |  |
| *β-ACTIN* | Forward | 5’-AGAGCTACGAGCTGCCTGAC-3’ | 80 | 56°C |
|  | Reverse | 5’-AGCACTGTGTTGGCGTACAG-3’ |  |  |

**Supplemental Table 2.** Characteristics of the flow cytometry antibodies used in this study.

| **Antibody** | **Fluorochrome** | **Clone** | **Manufacturer** |
| --- | --- | --- | --- |
| Mouse anti-CD3 | PE-Cy5 | UCHT1 | BD Biosciences |
| Mouse anti-CD4 | FITC | RPA-T4 | BD Biosciences |
| Mouse anti-CD25 | PE-Cy7 | BC96 | eBioscience |
| Mouse anti-CD127 | Horizon V450 | HIL-7R-M21 | BD Biosciences |
| Mouse anti-CD39 | APC | TU66 | BD Biosciences |
| Rat anti-FoxP3 | PE | PCH101 | eBioscience |
| Mouse anti-CD4 | PE-Cy7 | SK3 | BD Biosciences |
| Mouse anti-IFNγ | FITC | 4S.B3 | eBioscience |
| Mouse anti-TNF | APC | MAb11 | BD Biosciences |
| Mouse anti-IL-17A | PE | SCPL1362 | BD Biosciences |
